# Structural basis of RNA polymerase I pre-initiation complex formation and promoter melting

**DOI:** 10.1038/s41467-020-15052-y

**Published:** 2020-03-05

**Authors:** Michael Pilsl, Christoph Engel

**Affiliations:** Regensburg Center for Biochemistry, University of Regensburg, Regensburg, Germany

**Keywords:** Biochemistry, Electron microscopy, Molecular modelling

## Abstract

Transcription of the ribosomal RNA precursor by RNA polymerase (Pol) I is a prerequisite for the biosynthesis of ribosomes in eukaryotes. Compared to Pols II and III, the mechanisms underlying promoter recognition, initiation complex formation and DNA melting by Pol I substantially diverge. Here, we report the high-resolution cryo-EM reconstruction of a Pol I early initiation intermediate assembled on a double-stranded promoter scaffold that prevents the establishment of downstream DNA contacts. Our analyses demonstrate how efficient promoter-backbone interaction is achieved by combined re-arrangements of flexible regions in the ‘core factor’ subunits Rrn7 and Rrn11. Furthermore, structure-function analysis illustrates how destabilization of the melted DNA region correlates with contraction of the polymerase cleft upon transcription activation, thereby combining promoter recruitment with DNA-melting. This suggests that molecular mechanisms and structural features of Pol I initiation have co-evolved to support the efficient melting, initial transcription and promoter clearance required for high-level rRNA synthesis.

## Abstract (web summary)

RNA polymerase I (Pol I) catalyses the transcription of ribosomal RNA precursors, and its transcription initiation mechanism differs from that of Pol II and Pol III. Here the authors present the cryo-EM structure of a trapped early intermediate stage of promoter-recruited Pol I, which reveals the interactions of the basal rDNA transcription machinery with the native promoter, and discuss the mechanistic implications.

## Introduction

The transcription of the ribosomal RNA (rRNA) precursor by RNA polymerase (Pol) I is a prerequisite for ribosome biosynthesis in all known eukaryotes^1^. As such, Pol I transcription is tightly regulated, mostly at the level of pre-initiation complex (PIC) formation^2–6^. Whereas Pol II and Pol III use related initiation mechanisms, the processes underlying Pol I promoter recognition, PIC formation and DNA melting substantially diverge^7–9^. In bakers’ yeast *Saccharomyces cerevisiae*, a basal initiation system required for Pol I activity consists of the promoter DNA core element (CE), specific initiation factor Rrn3 and heterotrimeric core factor (CF)^10^. CF binds a CE stretch between ~15 and 38 base pairs (bps) upstream of the transcription start site (TSS)^11^ and recruits Rrn3-stabilized Pol I that is primed for initiation^12–14^. DNA melting occurs at a position slightly upstream of the TSS between the Pol I ‘clamp core’ and ‘protrusion’ domains^15–17^. No additional factors are required to commence initial transcription and promoter escape. In a complete system, however, upstream activating factor (UAF) recognizes an upstream element (UE) and cooperates with the TATA-binding protein (TBP) to stabilize CF association with the promoter, increasing Pol I initiation rates by up to 40-fold in vitro^18–21^. Furthermore, the factor Net1 may reside at Pol I promoters and has been described to enhance initiation in vivo and in vitro^22,23^.

During transcription initiation, Pols are recruited to their promoters by a set of general transcription factors, forming a ‘closed complex’ (CC). After melting of both DNA strands, an ‘open complex’ (OC) is established, transitioning into an ‘initially transcribing complex’ (ITC) with the beginning of RNA chain synthesis. In ITCs, a stable DNA/RNA hybrid is formed and the polymerase has initiated movement into the gene before establishment of a processive elongation complex (‘EC’; for a review of initiation phases compare refs. ^24,25^). Previous structural analyses of Pol I initiation complexes by us and others relied on an artificially stabilized, mismatched bubble scaffold assembled with an initially transcribed RNA sequence and a double-stranded DNA (dsDNA) sequence extending to up to 24 bps downstream of the TSS^15–17^. This experimental approach originates from the analysis of Pol II elongation complexes (ECs), preventing heterogenic sample conformations and making use of the tight DNA/RNA hybrid association with the polymerase^26,27^. In the case of the Pol I PIC a similar experimental strategy results in the visualization of late initiation intermediates. Consequently, an inconsistent occupancy of Rrn3 and divergent localization of the tandem-winged helix (twh) domain of Pol I subunit A49 and the C-terminal domain of subunit A12.2 have been observed^15–17^, leaving room for speculation with regard to the functional roles and temporal classification of the analyzed conformations during initiation^7–9^.

Therefore, we aimed at analyzing Pol I initiation mechanisms at an early initiation stage, allowing the visualization of promoter recognition, Pol I recruitment and DNA melting in a scenario as close to the native situation as possible. For this purpose, we assembled a complete initiation complex on double-stranded (ds) promoter DNA and performed single-particle cryo-EM analysis. The dsDNA scaffold was truncated on its downstream edge at position +8 relative to the TSS, thus preventing a contact with the clamp core and jaw domains of the polymerase. Three-dimensional particle reconstruction, cryo-EM density refinement and structural modeling allow the placement of basal PIC components and a comparative PIC analysis of the three eukaryotic Pols. Furthermore, structure-guided analysis indicates how Pol-I-specific ribosomal DNA (rDNA) promoter melting may be achieved.

## Results

### Complex formation and cryo-EM analysis

**Fig. 1:**
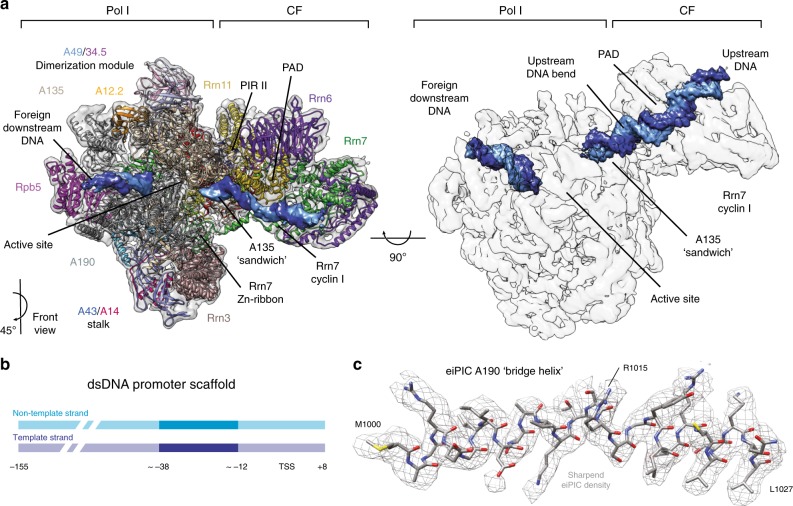
Cryo-EM reconstruction of a Pol I early intermediate PIC. **a** Overview of the Pol I eiPIC cryo-EM reconstruction at 3.5 Å resolution (unsharpened; transparent gray envelope) overlaid with the PDB model (colored ribbon) and DNA (space filing). The right panel shows transparent density (gray) for protein components and solid density for the DNA path (template strand in blue and non-template in light blue). PAD promoter-associated domain (of Rrn11); PIR polymerase interacting region (of CF). **b** Schematic representation of promoter dsDNA used for PIC assembly, densities observed in the eiPIC reconstruction are highlighted in blue and light blue for template strand and non-template strand, respectively. **c** Atomic model of the bridge helix in subunit A190 overlaid with sharpened eiPIC density (gray mesh) indicates residue orientations.

To study promoter recognition and DNA melting, we formed a complete Pol I initiation complex in vitro. UAF was assembled on a dsDNA promoter scaffold ranging from position −155 to +8 relative to the TSS together with TBP, CF, and a fragment of the protein Net1^22,28,29^ (Methods). Endogenously purified Pol I^13,30,31^ was pre-incubated with recombinant Rrn3^32^ to reconstitute a complete early PIC that was stable throughout size exclusion chromatography (Supplementary Fig. 1a; Methods). Accordingly, Pol I could be recruited to a UAF/TPB/Net1/CF-bound promoter scaffold lacking sequence stretches required for forming extended downstream contacts with the jaw- and clamp-head domains of the polymerase. Single-particle cryo-EM data was collected on a Titan Krios equipped with Gatan K2 summit direct electron detector basically as described^12,13^. Following pre-processing, two-dimensional (2D)- and three-dimensional (3D)-classification in RELION^33^, a total of 122,099 particles were selected from 4,088 micrograph movies (Methods; Supplementary Fig. 1). A final cryo-EM reconstruction exhibits an overall resolution of 3.5 Å and shows a Pol I early intermediate PIC (eiPIC; Fig. 1 and Supplementary Fig. 1). The cryo-EM density clearly reveals secondary structure features for the entire particle and side chain orientations in most regions (Fig. 1c and Supplementary Fig. 2a–f). Despite protein–protein crosslinking, TBP, UAF, and Net1-CTR remain flexible, although apparently stabilizing CF similar to the co-activator ‘mediator’ in context of a Pol II PIC^34^.

### An early intermediate PIC exhibits a well-defined architecture

Initial assignment located template and non-template DNA strands, Pol I, CF subunits, and Rrn3, followed by manual model building and real-space refinement, resulting in a model of high quality (Methods, Table 1). Upstream DNA is well-ordered between CF-interacting regions and entry into the Pol I active center cleft. Following the canonical DNA-path further downstream, however, no density is visible around the active center itself, but ≥12 well-defined base-pairs can be placed on the downstream edge between bridge helix and the clamp-head/jaw domains, even though our scaffold should not extend this far. Most likely the conserved^35^ and highly charged region is bound by foreign DNA or the far upstream end of our scaffold. A similar effect was observed for patches of the nucleosome, after transcription by Pol II ‘peeled’ off supercoiled DNA^36^. Well in line, in vitro initiation assays previously showed a strong preference for Pol I to initiate from dsDNA ends of synthetic sequences^15^.

**Table 1:** Cryo-EM data collection, refinement, and validation statistics.

	eiPIC EMDB-10544 PDB 6TPS	CF in eiPIC EMDB-10663
Data collection and processing		
Magnification	105,000	105,000
Voltage (kV)	300	300
Electron exposure (e–/Å^2^)	56	56
Defocus range (μm)	−1.5 to −3.1	−1.5 to −3.1
Pixel size (Å)	1.09 (0.545 superres)	1.09 (0.545 superres)
Symmetry imposed	C1	C1
Initial particle images (no.)	311,557	311,557
Final particle images (no.)	122,099	122,099
Map resolution (Å)	3.54	3.91
FSC threshold	0.143	0.143
Map resolution range (Å)	3.3 to 9.9	3.4 to 16.7
Refinement		
Initial model used (PDB code)	6TPS	
Model resolution (Å)	3.5	
FSC threshold	0.143	
Map sharpening *B* factor (Å^2^)	−75	
Model composition		
Non-hydrogen atoms	50,070	
Protein residues	6,109	
Ligands	8 (Zn and Mg)	
*B* factors (Å^2^)		
Protein	65.6	
Ligand	102.9	
R.m.s. deviations		
Bond lengths (Å)	0.009	
Bond angles (°)	0.985	
Validation		
MolProbity score	1.85	
Clashscore	5.96	
Poor rotamers (%)	0.59	
Ramachandran plot		
Favored (%)	91.10	
Allowed (%)	8.75	
Disallowed (%)	0.15	

Initiation factor Rrn3 is tightly bound to Pol I ‘stalk’ and ‘dock’ subdomains^12,13^ in all analyzed particles, agreeing with chromatin immuno precipitation (ChIP) and biochemical studies in yeast^2,32,37^ and mouse^38,39^ cells. CF is associated with the Pol I core via its polymerase interacting regions (PIR) similar to ITC conformations^15–17^. Excellent quality of the cryo-EM density allowed us, to rebuild the CF subunits Rrn6, Rrn7 and Rrn11, consolidating divergent assignments in the crystal structure^15^ (PDB 5O7X) and an ITC EM-based model^17^ (PDB 5W66). In contrast to inactive Pol I^30,31,40^, the ‘expander’ and ‘connector’ subdomains are flexible and the central bridge helix is refolded in the eiPIC (Fig. 1c) as expected from EC structures^41,42^. The C-terminal domain of subunit A12.2 shows only residual density in funnel domain of subunit A190 (Supplementary Fig. 2b), but is not localized on the A135 lobe as observed in a 12-subunit EC^43^. Our eiPIC reconstruction shows strong density for the A49/A34.5 dimerization and A34.5 C-terminal tail domains (Supplementary Fig. 2e), indicating that the heterodimer is constitutively attached. The twh and linker domains of subunit A49 are detached in the eiPIC, agreeing with a proposed role in promoter escape^17^.

### Core factor embraces the promoter DNA

The eiPIC density allows the construction of a CF model, which we found to resemble the overall ITC conformation. To define the structural changes that take place upon promoter recruitment, we compared the architecture of CF in free (PDB 5O7X) and promoter-engaged eiPIC conformation (Supplementary Fig. 3). This shows that CF module I and II retract from each other by up to 12 Å upon binding of the CE promoter sequence. This retraction leads to the exposure of positively charged residues that are now free to engage the phosphate backbone (Supplementary Fig. 3a–c). These DNA-binding regions lie within the Rrn11 promoter-associated domain (‘PAD’) and the cyclin domains of Rrn7. The same regions engage the DNA in ITCs^15–17^ and have been described in detail in late ITCs devoid of Rrn3^17^. Remarkably, the Rrn7 residues involved in DNA-binding are not conserved within TFIIB or Brf1, which share a similarity in their overall fold^44–46^ and would clash with TBP^15^ in canonical TFIIB-TBP^47^ or Brf1-TBP^48,49^ complex.

**Fig. 2:**
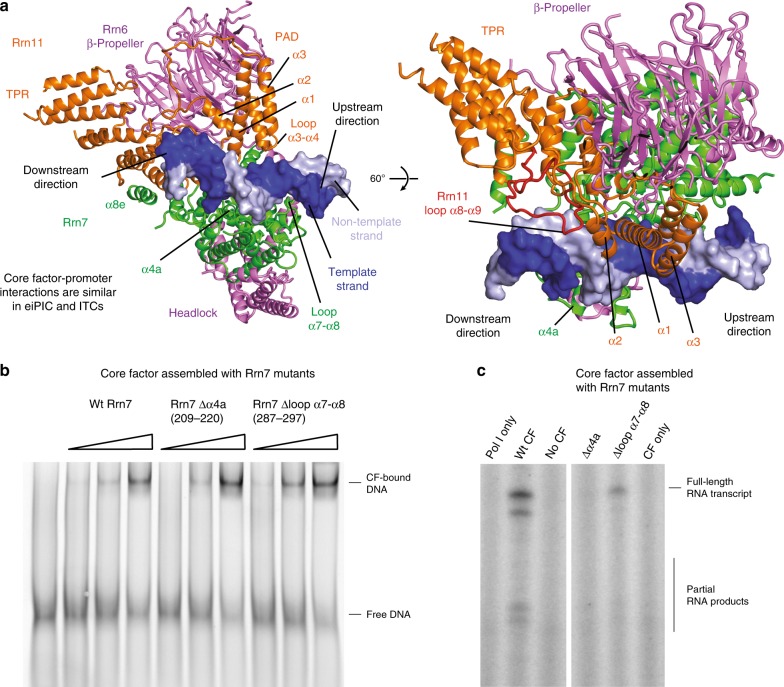
Core Factor—promoter interactions in eiPIC. **a** Model of promoter-bound CF in the eiPIC. The same regions of Rrn7 and Rrn11 contribute to promoter phosphate backbone interactions compared to ITC reconstructions. **b** Electrophoretic mobility shift assay (EMSA) shows that wild-type CF interacts with double-stranded promoter DNA (0.25 pmol, 0.5 pmol, and 1 pmol CF added). Mutation of Rrn7 (Δα4a and Δloop α7-α8) does not impair promoter-DNA association. **c** In contrast to DNA binding, initiation efficiency of CF assembled with Rrn7 mutants Δα4a and Δloop α7-α8 is impaired (promoter-dependent in vitro transcription assay from a minimal scaffold).

Comparison of free and promoter-engaged CF also shows that the Rrn7-specific helix α4a in the N-terminal cyclin domain shifts and is inserted into the minor groove of the CE promoter DNA, while loop α7-α8 in cyclin II becomes well-structured and contacts the major groove further upstream upon eiPIC formation (Fig. 2a). Thereby, the distal upstream DNA-path is modified towards the C-terminal domain of Rrn7 and the β-propeller-domain of Rrn6. Thus, promoter binding by Rrn7-specific regions on one face and by the TFIIB-unrelated CF subunit Rrn11 on the opposite face tightly squeeze the DNA. This may explain why the basal Pol I initiation system does not require TBP association opposite of the Rrn7 cyclins.

To address the importance of these residues, we constructed CF mutants with deletions in helix α4a and in loop α7-α8. Both can still associate with promoter DNA (Fig. 2b), but show defects in basal initiation in vitro (Fig. 2c). Engagement of these regions may therefore be important to induce a specific DNA conformation required for Pol I recruitment or promoter melting.

### The Pol I ‘sandwich’ region is important for PIC formation

We have previously described a Pol-I-specific proximal upstream promoter-binding region consisting of loop α11a-α12 (residues 452–456) and the loop β28-β28 (residues 815–818) in the protrusion and wall domains of Pol I subunit A135, respectively^15^. In the eiPIC, a positively charged loop (892–895, wall domain of subunit A135) re-orients towards the promoter DNA, contributing additional phosphate-backbone interactions (Supplementary Fig. 4) similar to other ITC/PIC structures^16,17^. These promoter interactions are all specific to Pol I, because the residues are not conserved in Pol II^50^ and III^51^. Furthermore, DNA is occluded from the corresponding region in Pol II and III PICs by the N-terminal cyclin domains of TFIIB^34,52,53^ and Brf1/Brf2^48,49,54,55^, respectively. Fittingly, this Pol I region was previously named ‘sandwich’^17^.

In the eiPIC, the sandwich region tightly holds the promoter in place between the wall and protrusion domains at the bottom of the cleft. sandwich elements contact both DNA strands, therefore rendering it specific for an un-melted duplex. Density for the DNA directly downstream of the sandwich is not observed, indicating a higher degree of flexibility. Consequently, the recruitment of the Pol-I-Rrn3 complex seems to mainly rely on (1) contacts between the promoter and the sandwich and (2) protein–protein contacts between CF and the Pol-I-Rrn3 complex. In contrast, further promoter contacts with the Pol I cleft or downstream elements and/or A49 appear not to be required for recruitment.

### TFIIB-related elements in Rrn7 adopt divergent positions

**Fig. 3:**
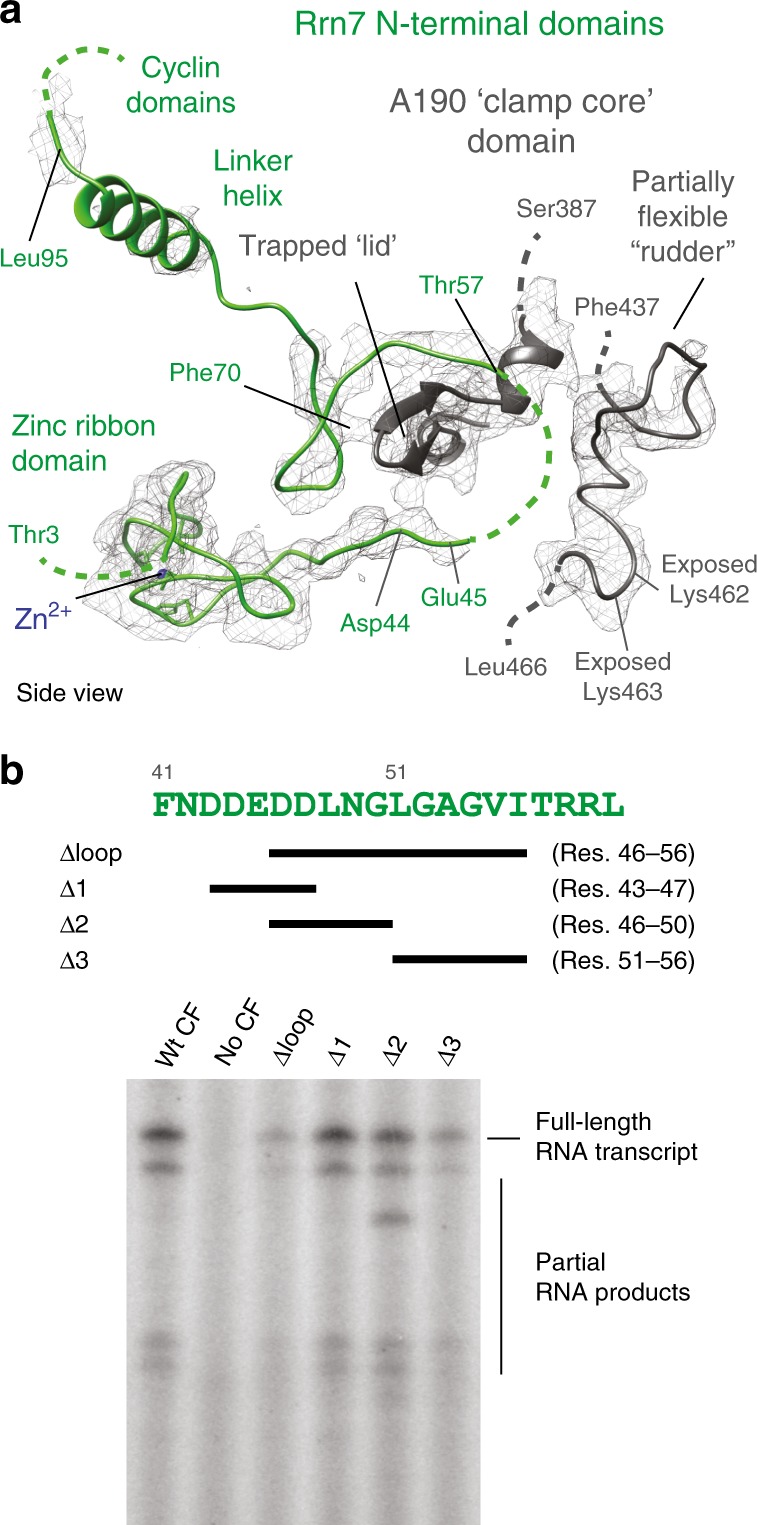
The N-terminal region of Rrn7 is partially ordered within the eiPIC. **a** Ribbon model of TFIIB-homologous regions in the N-terminus of Rrn7 (green) overlaid with sharpened eiPIC density (gray mesh). The lid domain of Pol I subunit A190 (dark gray ribbon) is trapped between well-ordered regions of Rrn7. Residues 46 to 56 of Rrn7 are partially flexible, hinting at a function during promoter melting. **b** Amino acid sequence of flexible Rrn7 region is shown in green with schematic representation of deletion mutants indicated by black bars. Basal in vitro initiation assay shows the effect of Rrn7 mutations within this loop: Deletion of the entire loop and its C-terminal part (51 to 56) show reduced initiation activity.

The TFIIB-related ‘reader’ and ‘linker’ elements within Rrn7^44,46^ are mostly ordered in the active center cleft of the eiPIC, with the exception of the residues 46–56 (B-reader homologous^56^). The protein backbone extends from the N-terminal zinc ribbon into the Pol I cleft, apparently trapping the well-ordered ‘lid’ subdomain of Pol I subunit A190 before forming two anti-parallel strands and exiting the Pol I upstream face on the side of the shelf module (Fig. 3a). The path of Rrn7 differs from a Pol I ITC^17^ and from TFIIB in complex with Pol II^57^ (Supplementary Fig. 5). During Pol II initiation, the TFIIB-reader-loop contacts the ‘rudder’ and the ‘fork loop I’ domains, while the TFIIB-linker binds the top of the rudder and forms a helix that interacts with the clamp core domain^57^. In the eiPIC, rudder and fork loop I apparently interact neither with each other nor with the TFIIB-reader-homologous regions of Rrn7. Instead, rudder and fork loop I are oriented towards the bridge helix and an Rrn7 helix that is similar to the TFIIB linker connects to CF module II.

In addition to a divergent path of Rrn7 compared to TFIIB, the residues contacting the template strand in a Pol I ITC^17^ and Pol II ITC^57^ are mostly flexible in the eiPIC, but not in Pol II CCs^52^ or in a Pol II-TFIIB^57^ complex. Furthermore, TFIIB reader-loop arginine residue 78, which is important for TSS selection by Pol II^58^, does not exist in Rrn7. This adds to overall sequence^44,46^ and architecture differences^15^ between Rrn7 and TFIIB.

To clarify the importance of Rrn7 loop residues disordered in the eiPIC, we mutated the entire loop or smaller stretches and analyzed CF initiation activity in a basal assay (Fig. 3b). The loop-deletion Rrn7 mutant shows strongly reduced initiation efficiency, which can mainly be attributed to the residues 51–56, but not to residues 43–50. The Rrn7 version with loop-deletion still assembles well with Rrn6 and Rrn11 and is able to form a basal PIC in vitro (Supplementary Fig. 3d, e). Thus, the Rrn7-reader-loop is likely important for promoter melting.

### Pol I is primed for initiation at the eiPIC stage

**Fig. 4:**
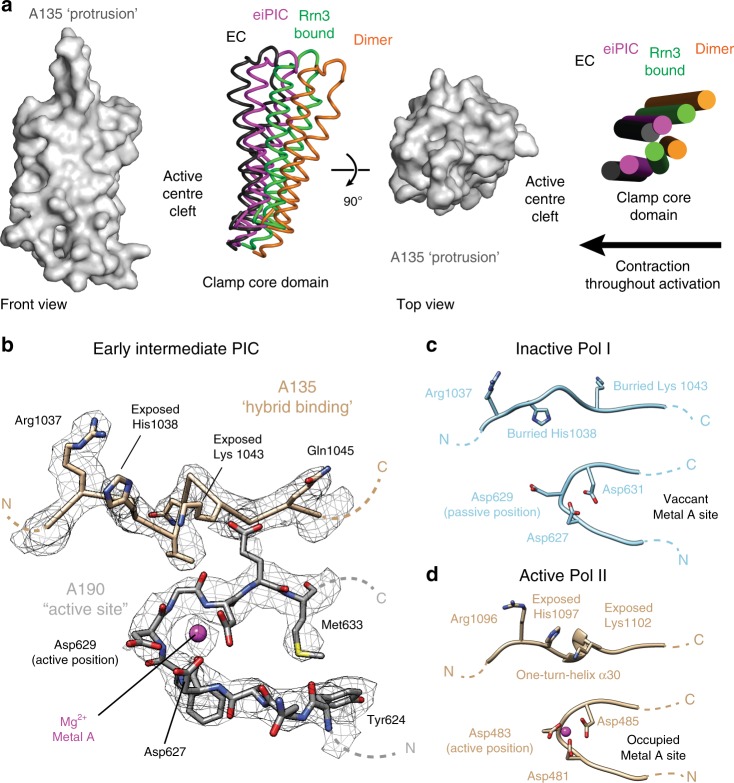
Pol I is primed for initiation in the eiPIC. **a** Cleft contraction throughout activation stages. Pol I structural models were overlaid via their A135 subunits (protrusion subdomain in gray, space filling). Cleft contraction is indicated by colored clamp core helices (subunit A190). Monomeric Pol I and ITC stages are similar to Rrn3-bound- and EC-conformations, respectively (not shown for clarity). PDB models displayed: 4C2M (orange), 5G5L (green), eiPIC (magenta) and 5M3F (black). **b** Atomic model of the active center and hybrid-binding domains within Pol I subunits A190 and A135, respectively. Overlaid with sharpened eiPIC density (gray mesh). The metal A site is occupied and a one-turn-helix α30 is formed in A135, exposing positively charged residues. **c** Inactive Pol I (PDB 4C2M) region for comparison to **b**. **d** Active Pol II region for comparison to **b** and **c**.

Modeling of the active center based on our eiPIC density indicates, that aspartate 629 in subunit A190 (Asp483 in Pol II subunit Rpb1) has apparently changed its orientation with respect to the dimeric crystal structures^30,31^ (Fig. 4). Assuming its active orientation in the eiPIC, Asp629 now allows coordination of the catalytic magnesium ion (‘metal A’), together with Asp627 and Asp631 for which we observe a clear cryo-EM density peak (Fig. 4b). In addition, the hybrid-binding domain of subunit A135 re-arranges to form a one-turn helix in the eiPIC. This helix also resembles the active Pol I, II, and III EC conformations and its formation exposes histidine 1038 to the bottom of the cleft, which is now free to contact the hybrid upon initial transcription as observed in ITCs. Furthermore, the previously buried lysines 462 and 463 in subunit A190 become exposed in the eiPIC (Supplementary Fig. 2f), now resembling the active Pol-II-fold^59^ and contacting the first visible downstream DNA base pair. This may contribute to a high affinity for foreign DNA and to the Pol I preference for initiation from ends of dsDNA. With the described structural changes upon eiPIC formation, Pol I enters a conformation that is primed for initial transcription via a conserved mechanism^60^ in the presence of NTPs.

We also observe, that the Pol I cleft continues to contract downstream of the sandwich region, adapting an intermediate conformation between the Rrn3-bound and ITC/actively elongating states (Fig. 4a). This adds an additional intermediate to the set of Pol I structures^9^, but is in line with the suggestion, that cleft modulation is a major regulatory mechanism of Pol I transcription^14,30,61^. At the stage of DNA-melting during the transition from CC to OC states, dsDNA cannot be accommodated between clamp core and protrusion domains any longer^15^. Hence, simultaneous promoter loading and cleft contraction allosterically destabilize the upstream duplex at the position of the clamp core and may foster spontaneous melting at this position. Notably, the initially melted region shows the highest conservation among rDNA promoters identified thus far^62^. Thus, the eiPIC apparently represents a trapped CC-OC transition intermediate conformation, which is important for spontaneous DNA-melting to take place during promoter association of the polymerase.

## Discussion

Within this work, we describe an early intermediate initiation complex. The structure enables the independent discussion of promoter recruitment and DNA-melting in a sequential manner. Apparently the polymerase is recruited to its dsDNA promoter but cannot complete the melting process due to a lack of fixated downstream DNA. We described the eiPIC reconstruction in the context of PIC formation and continue to update our model of Pol I recruitment and DNA-melting in light of these findings. Our interpretation is well in line with the idea that targeting of the initiation machinery to the rDNA promoter depends mostly on UAF, and TBP serves to position CF downstream of the UE, while interacting with the promoter using a divergent interface^63^. Recruitment of the Pol-I-Rrn3 complex then relies on a specific DNA architecture^64^, namely a bendability that allows interactions of the Rrn11 TPR domain with the Pol I protrusion^15^ and binding of a promoter element to the Pol I sandwich region (Supplementary Fig. 4). Since our assembly originally comprised UAF and TBP, and only a single reconstruction was obtained from 39% of all recorded particles, it is likely that we capture a physiologically relevant conformation, while factors were artificially positioned by DNA/RNA hybrid scaffolds simulating initial transcription in previous analyses^15–17^, even though RNA was lost in one case^16^.

Within the eiPIC structure, re-arrangements between CF module I and II enable Rrn7 and Rrn11 to bind promoter DNA, mainly by phosphate backbone interactions of basic loops. This explains the (low) sequence specificity of DNA-binding by CF and thus the overall similar eiPIC architecture compared to ITCs and late PIC reconstructions. Likely, Rrn7-specific DNA-interacting loops contribute to DNA-conformational modulation (compare Fig. 2). We further confirm cleft contraction between the protrusion and clamp core domains and exposure of basic residues bottom of the cleft during DNA-melting by Pol I in the eiPIC.

While our findings do not oppose the idea of an upstream ratchetting mechanism to open Pol I promoter DNA, we also see no evidence to support such a mechanism deduced from shifts in CF-positions observed in ITC reconstructions^17^.

Instead, we propose a simplified melting-mechanism based on steric DNA-distortion and electrostatic single-strand trapping which, in this combination, is only possible in Pol I, but not in Pol II and III. Firstly, Pol I recruitment relies on DNA-duplex binding to the sandwiching region and DNA positioning within the expanded cleft of the Pol-I-Rrn3 complex (Fig. 4a and Supplementary Fig. 4). Sequence specificity is determined by proximal upstream bendability^15,65^ and distal upstream recognition by UAF, which is linked to the PIC via CF and TBP. Divergent TFIIB reader-loop elements within Rrn7 are placed in the Pol I cleft, may play a role in duplex-destabilization and bind the melted template strand similar to observations in ITCs^17^. In addition, allosteric duplex-destabilization resulting from a cleft contraction between the clamp and protrusion domains observed in the eiPIC likely contributes to melting (Fig. 4a). This contraction primes Pol I for initial transcription by re-ordering previously inactivated regions (Figs. 1 and 4 and Supplementary Fig. 2). Exposed basic residues can then contribute to stabilization of the initially melted template strand and ultimately the DNA/RNA hybrid at the bottom of the cleft. Furthermore, the non-template strand may be bound by the A49 linker (as observed in ref. ^17^), thereby preventing collapse of the early bubble similar to the σ-factor in bacterial Pol^66,67^. Only after initial transcription, the growing RNA chain can interact with Rrn7 and would finally clash with reader/linker elements, freeing the exit channel and expelling Rrn7 from the polymerase. This is probably concerted with the association of the flexible A49 twh domain at the back of the clamp core domain, leading to dissociation of CF and Rrn3 and preventing re-association, thereby fostering promoter escape.

In Pol II and Pol III initiation complexes^48,49,53,68^, TFIIB/Brf1 cyclin domains occlude the sandwiching region and reader/linker domains diverge from Rrn7, preventing a similar mechanism. Arguing for a model of combined adaptations, a number of CF-mutations impaired in vitro initiation rates, but only large deletions completely abolished functionality^15,45^. Furthermore, a 12-subunit Pol I lacking A49/A34.5 is still able to initiate from its native promoter (although the lack of A49 linker-positioning strongly impaired the process)^13,37^, TBP is not necessary for basal transcription^11,21^ and single A49 mutations have only minor effects on Pol I function^69^. Thus, the overall functionality of the system is robust and highly adaptive to conditional variations. However, full initiation rates required for physiological growth depend on the combined action of all Pol-I-specific elements that have accumulated throughout evolutionary adaptation and are basically conserved throughout eukaryotic organisms^10,70,71^. These adaptations increase initial transcription to such efficiency, that formation of a stable closed complex under physiological conditions appears unlikely. While such a state may be transiently established, the instant cleft contraction and Rrn7-dependent duplex-destabilization by the combined action of Pol I and CF elements directly lead to melting and prime the polymerase for initial transcription and hybrid stabilization.

During the final stages of revision of this work, a related study was published^72^. Sadian et al. provide an excellent description of CF-promoter contacts in detail and investigate the role of an acidic loop in Rrn3, based on higher resolution reconstructions. Compared to our results, interpretation relies on a minor subset of 0.7% or 0.5% of particles from two datasets indicating a transient nature of CCs. In our UAF/TBP-containing samples, however, 39% of initial particles contribute to the final reconstruction and divergent CF-positions are not observed. This may be due to a lack of available particles in our datasets, or due to stabilization of a more ‘native’ CF-orientation in the presence of UAF/TBP. Detailed structure-function analysis of UAF- and TBP-contributions are now instrumental to understanding the process of Pol I initiation in its entirety.

## Methods

### Protein expression and purification

Partially purified endogenous *Saccharomyces cerevisiae* Pol I is a by-product of Pol III purification via a TAP-tag on subunit AC40^49^. The Pol-I-containing MonoQ fractions were a gift from A. Vannini and G. Abascal-Palacios. Fractions were pooled, diluted fivefold in buffer A (20 mM HEPES/KOH pH 7.8, 10% glycerol, 1 mM MgCl_2_, 10 µM ZnCl_2_, 5 mM dithiothreitol (DTT)) and loaded onto a MonoS HR 5/5 column (GE Healthcare). Separation was performed with a gradient from 10–37,5% buffer B (buffer A with 2 M KAc) with a 2 CV plateau at 17,5% B. Pol I eluted at 470 mM KAc^73^, peak fractions were pooled, flash frozen in liquid nitrogen and stored at −80 °C.

Rrn3^32^ was expressed in BL21(DE3) pRIL (Agilent) cells, by autoinduction in TB medium (1.2% tryptone; 2.4% yeast extract; 0.5% glycerol); 1/10 volume of a sterile solution containing 0.17 M KH2PO4 and 0.72 M K2HPO4 and 1/50 volume of a sterile solution containing 25% glycerol; 10% lactose and 1% glucose were added. A culture was grown at 37 °C to an OD_600_ of 0.6, after cooling the culture on ice, incubation was continued at 16 °C overnight. Cells were harvested (6000 g; 10 min), resuspended in lysis buffer (50 mM HEPES at pH 7.8, 200 mM NaCl, 3 mM DTT, 10% glycerol). A 3 ml Ni-NTA column (Qiagen) was equilibrated with lysis buffer, the supernatant loaded, and the column was washed with lysis buffer containing 25 mM imidazole. Elution was carried out in lysis buffer containing 150 mM imidazol. Next, Rrn3 was further purified by anion exchange chromatography (Mono Q 5/50, GE Healthcare). The column was equilibrated in MonoQ buffer 1 (50 mM HEPES at pH 7.8, 5 mM DTT, 10% glycerol), and proteins were eluted with a linear gradient of 20 column volumes from 100 mM to 1 M NaCl. After concentration (Amicon, 35 kDa cutoff), the sample was applied to a Superdex 200 increase 10/300 size exclusion column (GE Healthcare) equilibrated with buffer Rrn3-SEC (20 mM HEPES at pH 7.8, 300 mM NaCl, 5 mM DTT).

CF subunits^15^ were co-expressed in *E. coli* BL21-CodonPlus(DE3)-RIL cells (Agilent) from two plasmids. A 4 l culture was grown in LB medium at 37 °C until OD600 reached 0.5–0.7. Cultures were cooled on ice for 20 min and expression was induced with 0.1 mM IPTG. Cells were grown at 18 °C overnight. Cells were harvested by centrifugation, washed with phosphate-buffered saline (PBS) at 4 °C, flash frozen in liquid nitrogen and stored at −80 °C. One pellet was suspended in buffer CF-A (20 mM imidazole, 350 mM NaCl, 10 mM MgCl_2_, 10% (v/v) glycerol, 20 mM HEPES pH 7.8, 1 mM DTT, 1x protease inhibitor). Cells were lysed by sonication using a Branson Digital Sonifier, the lysate was cleared by centrifugation and the supernatant was filtered with a 0.22 µm filter (Millipore) to remove cell debris. Cell lysate was then applied to a Ni-NTA column (5 ml, GE Healthcare) and bound CF washed with 5 CV of buffer CF-B (25 mM imidazole, 200 mM NaCl, 10 mM MgCl_2_, 10% (v/v) glycerol, 20 mM HEPES pH 7.8, 1 mM DTT) at 4 °C. The column was transferred to room temperature, washed with 2.5 CV of buffer CF-C (50 mM imidazole, 200 mM NaCl, 10 mM MgCl_2_, 10% (v/v) glycerol, 20 mM HEPES pH 7.8, 1 mM DTT, 5 mM ATP, 2 mg/ml denatured protein), incubated for 10 min, and washed again with 2.5 CV buffer CF-C. The column was transferred to 4 °C and washed with 5 CV buffer CF-D (50 mM imidazole, 200 mM NaCl, 10 mM MgCl_2_, 10% (v/v) glycerol, 20 mM HEPES pH 7.8, 1 mM DTT). Elution was performed with 5 CV of buffer CF-E (350 mM imidazole, 200 mM NaCl, 10 mM MgCl_2_, 10% (v/v) glycerol, 20 mM HEPES pH 7.8, 1 mM DTT). Protein was then loaded on a 5 ml heparin column (GE Healthcare) in buffer CF-F (200 mM NaCl, 1 mM MgCl_2_, 10% (v/v) glycerol, 20 mM HEPES pH 7.8, 1 mM DTT) and eluted with a gradient ranging from 0.2 to 2.0 M NaCl, including a plateau at 550 mM NaCl of 2 CVs. CF-containing fractions were concentrated using a 100 kDa cutoff centrifugal filter (Millipore). Size exclusion chromatography was carried out with a Superose 6 increase 10/300 column (GE Healthcare) in buffer CF-G (200 mM NaCl, 1 mM MgCl_2_, 5% (v/v) glycerol, 10 mM HEPES pH 7.8, 10 μM ZnCl2, 1 mM DTT). CF-containing fractions were concentrated using a 100 kDa cutoff centrifugal filter (Millipore) and directly used or flash frozen in liquid nitrogen for storage at −80 °C.

*S. cerevisae* TBP was cloned into vector pET28b via NheI/Not I restriction sites (compare Supplementary Table 1). Recombinant His_6_-TBP protein was expressed in BL21(DE3) pRIL (Agilent) cells, by autoinduction in TB medium (1.2% tryptone; 2.4% yeast extract; 0.5% glycerol; 1/10 volume of a sterile solution containing 0.17 M KH2PO4 and 0.72 M K2HPO4 and 1/50 volume of a sterile solution containing 25% glycerol; 10% lactose and 1% glucose were added. A culture was grown at 37 ° C to an OD_600_ of 0.6, after cooling the culture on ice, incubation was continued at 16 ° C overnight. Cells were harvested (6000 g; 10 min), resuspended in lysis buffer (50 mM HEPES/KOH; 10% glycerol; 10 mM MgAc_2_; 200 mM KCl; 10 mM imidazole; 5 mM β-mercaptoethanole; 1 mM phenylmethylsulphonyl fluoride (PMSF); 2 mM benzamidine), and lysed by sonication (Branson Sonifier 250 macrotip, cooling in icewater). The cell extract was cleared twice (40,000 g for 40 min at 4 °C) and incubated with 1 ml equilibrated NiNTA Agarose (Qiagen) at 4 °C for 2 h on a rotating wheel. The resin was transferred to a polypropylene column (Bio-Rad), washed with wash buffer 1 (20 mM HEPES/KOH; 10% glycerol; 5 mM MgAc_2_; 1 M KCl; 20 mM imidazole; 5 mM b-mercaptoethanole), wash buffer 2 (as wash buffer 1 but with 0.2 M KCl) and eluted with elution buffer (20 mM HEPES/KOH; 10% glycerol; 5 mM MgAc_2_; 0,2 M KCl; 200 mM imidazole; and 5 mM b-mercaptoethanole). The sample was diluted with buffer C (20 mM HEPES/KOH pH 7.8, 10% glycerol, 1 mM MgCl_2_, 5 mM DTT) to ≈100 mM KCl and loaded onto a MonoS 5/50 GL column (GE Healthcare) and eluted with a linear gradient from 10–100% buffer D (buffer C + 1 M KCl). TBP containing fractions were pooled, concentrated and loaded onto a Superdex 75 10/300 Increase column (GE Helthcare) equilibrated in buffer E (20 mM HEPES/KOH pH 7.8, 10% glycerol, 200 mM KCl, 1 mM MgCl_2_, 5 mM DTT) peak fractions were pooled, flash frozen in liquid nitrogen and stored at −80 °C.

Net1-CTR-TAP was expressed in baculovirus infected SF21 cells as recently published^22,74–76^. Specifically, 50 × 10^6^ cells were resuspended in buffer F (20 mM HEPES/KOH pH 7.8, 10% glycerol, 200 mM KCl, 1 mM MgCl_2_, 0.2% NP40, 1 mM DTT), lysed by sonication (Branson Sonifier 250 macrotip, cooling in icewater) and cleared by centrifugation (40,000 × *g* for 40 min at 4 °C). The supernatant was incubated four hours with 1 ml IgG Sepharose 6 Fast Flow (GE Healthcare), washed with buffer F and incubated with TEV protease for two hours at 16 °C. Eluate fractions were collected, flash frozen in liquid nitrogen and stored at −80 °C. The MultiBac system was also used to generate a bacculo virus co-expressing Rrn5-HA, Rrn9-Flag, Uaf30-His7, Rrn10, Histones H3 and 4 in SF21 cells (Supplementary Table 1). In all, 1 × 10^9^ cells were resuspended in lysis buffer (50 mM HEPES/KOH pH 7.8, 10% glycerol, 400 mM (NH_4_)_2_SO_4_, 10 mM MgCl_2_, 20 mM imidazole, 1 mM DTT) and lysed by sonication (Branson Sonifier 250, cooling in icewater). The cleared lysate (2 × 70,000 g, 45 min, 4 °C) was incubated with 2 ml Ni-NTA Agarose (Qiagen) for 2 h, beads were washed with buffer G (20 mM HEPES/KOH pH 7.8, 10% glycerol, 1 M KCl, 5 mM MgCl_2_, 20 mM imidazole, 1 mM DTT), buffer H (20 mM HEPES/KOH pH 7.8, 10% glycerol, 400 mM KCl, 2 mM MgCl_2_, 50 mM imidazole, 1 mM DTT) and eluted with buffer I (20 mM HEPES/KOH pH 7.8, 10% glycerol, 400 mM KCl, 2 mM MgCl_2_, 300 mM imidazole, 1 mM DTT). The eluate was diluted with buffer C, loaded onto a MonoS HR 5/5 column and eluted with a gradient to buffer D (see above). Eluate fractions were collected, concentrated, flash frozen in liquid nitrogen and stored at −80 °C. Raw SDS-PAGE gels are shown in Supplementary Fig. 6.

### Promoter-dependent in vitro transcription

Promoter-dependent in vitro transcriptions were performed following our previously published protocols^13,15^ on core promoter scaffolds from position −38 to +24 relative to the TSS (Supplementary Table 1). Specifically, Promoter-dependent in vitro transcription reactions were performed as follows: A total of 50 ng template dsDNA template were used for each transcription reaction (25 μl reaction volume. CF was added to a final concentration of 20 nM pre-incubated Pol-I-Rrn3 complex was added to a final concentration of 4 nM. 20 mM HEPES/KOH pH 7.8 and 2 M KAc were added to adjust volume and salt concentration to the final reaction conditions of 150 mM KAc in 25 µl. Transcription was started by the addition of 5 μl 5x transcription buffer (100 mM HEPES/KOH pH 7.8, 50 mM MgCl_2_, 25 mM EGTA, 0.25 mM EDTA, 3 mM DTT, 1 mM ATP, 1 mM UTP, 1 mM CTP, 0.05 mM GTP complemented with 0.3 µl [α-32P]GTP (10 mCi/ml; Hartmann Analytic). The samples were incubated at 24 °C for 30 min. Next, 200 μl Proteinase K buffer (0.5 mg/ml Proteinase K in 0.3 M NaCl, 10 mM Tris/HCl pH 7.5, 5 mM EDTA and 0.6% SDS) was added to stop transcription. The samples were incubated at 56 °C for 15 min. Ethanol (700 μl) was added to allow precipitation of nucleic acids (30 min at −80 °C). The samples were centrifuged for 10 min at 12,000 × *g*, the supernatant was removed and the precipitate was washed with 150 µl 70% ethanol. After centrifugation, the supernatant was removed and the pellets were dried at 95 °C for 1 min. RNA in the pellet was dissolved in 12 μl 80% formamide, 0.1 M TRIS-Borate-EDTA (TBE), 0.02% bromophenol blue and 0.02% xylene cyanol. Samples were heated for 2 min under vigorous shaking at 95 °C and briefly centrifuged. After separation on a 20% polyacrylamide gel containing 8 M urea and 1x TBE. Radiolabelled transcripts are visualized using a PhosphoImager (GE Healthcare). Raw gels are shown in Supplementary Fig. 6.

### Electrophoretic mobility shift assays (EMSA)

For EMSA experiments, a fluorescently labeled promoter fragment (−83 to +26 relative to the TSS) was annealed from oligonucleotides labeled with fluorescent dyes (NTS position −3 Atto647N and TS position −5 Cy3) as described below. 0.2 pmol dsDNA was incubated for 30 min without or with increasing amounts (0.25, 0.5, 1 pmol) of recombinant mutant CF in incubation buffer containing 20 mM HEPES/KOH pH 7.8, 10% glycerol, 200 mM KCl, 1 mM MgCl_2_, 0.1 mg/ml BSA, 1 mM DTT. The reaction was loaded on a pre-run 6% native acrylamide gel in 0.5x TBE buffer and imaged on a Typhoon FLA 9000 (GE Healthcare) imaging system. Raw gels are shown in Supplementary Fig. 6.

### Pol I PIC assembly

The Pol I PIC was assembled on complementary rDNA promoter oligonucleotides AGCTTAAATTGAAGTTTTTCTCGGCGAGAAATACGTAGTTAAGGCAGAGCGACAGAGAGGGCAAAAGAAAATAAAAGTAAGATTTTAGTTTGTAATGGGAGGGGGGGTTTAGTCATGGAGTACAAGTGTGAGGAAAAGTAGTTGGGAGGTACTTCATGCGAAA (NTS), TTTCGCATGAAGTACCTCCCAACTACTTTTCCTCACACTTGTACTCCATGACTAAACCCCCCCTCCCATTACAAACTAAAATCTTACTTTTATTTTCTTTTGCCCTCTCTGTCGCTCTGCCTTAACTACGTATTTCTCGCCGAGAAAAACTTCAATTTAAGCT (TS) (Integrated DNA Technologies). Oligonucleotides were dissolved in TE buffer (10 mM Tris pH 8, 0.5 mM EDTA), mixed in equimolar amounts to a final concentration of 10 µM each, heated to 95 °C and slowly cooled down to 10 °C with a cooling rate of 1 °C/min.

In all, 0.11 nmol promoter DNA was incubated with equimolar amounts of UAF, and threefold molecular excess of TBP and Net1-CTR. After 20 min incubation at 28 °C, 0.17 nmol CF was added and incubated for additional 20 min. 0.095 nmol Pol I, pre-incubated overnight with fivefold molar excess of Rrn3 on ice, was added and the sample was diluted with buffer G (20 mM HEPES/KOH pH 7.8, 2 mM MgCl_2_, 5 mM DTT) to final assembly conditions (20 mM HEPES/KOH pH 7.8, 50 mM KAc, 50 mM KCl, 2 mM MgCl_2_, 10 µM ZnCl_2_, 5 mM DTT; buffer H), incubated further 30 min and concentrated to 50 µl. The sample was crosslinked with 1 mM (bis(sulfosuccinimidyl) suberate) (BS3) for 30 min at 28 °C. The Crosslinking reaction was quenched with 100 mM NH_4_HCO_3_ final concentration for 15 min at 28 °C. The sample was loaded onto a Superose 6 PC 3.2/30 column (GE Healthcare) equilibrated with buffer H (20 mM HEPES/KOH pH 7.8, 50 mM KAc, 50 mM KCl, 2 mM MgCl_2_, 10 µM ZnCl_2_, 5 mM DTT) and collected in 60 µl fractions. A raw sodium dodecyl sulfate–polyacrylamide gel electrophoresis (SDS-PAGE) gel of non-crosslinked sample is shown in Supplementary Fig. 6.

### Cryo-EM sample preparation and data acquisition

Grids were glow discharged in Argon/Oxygen plasma 90/10 (Fischione) for one minute. Four microliters of sample was applied to a grid (Quantifoi R 2/1 + 2 nm carbon, Quantifoil), incubated for 30 s, blotted 4 s with blot force ‘8’, at 100% humidity and 4 °C in a Vitrobot Mark IV (FEI) and plunged in liquid ethane.

Images were collected on a Cs-corrected Titan Krios microscope (FEI), operated at 300 kV using the multi-shot feature of the serialEM software^77^ for automated data collection. Movie frames were acquired on a 4k × 4k Gatan K2 summit direct electron detector in super-resolution mode at a nominal magnification of 105,000, which yielded a pixel size of 0.545 Å. Forty movie frames were recorded at a dose of 1.4 electrons per Å^2^ per frame corresponding to a total dose of 56 e/Å^2^.

### Image processing

Movie frames were aligned, dose-weighted, binned by a factor of 2 and averaged using MotionCor2^78^. Contrast Transfer Function (CTF) parameters were estimated with the Gctf ^79^ program. The RELION 3-beta suite^33^ was used for the whole-image processing workflow unless stated otherwise. The dataset was divided into four subsets with ~1000 images each. In a first step the reference-free auto-picking procedure based on a Laplacian-of-Gaussian (LoG) filter was used to identify ~100,000 starting coordinates (per subset), which were used to extract particles with threefold binning in a 140 pixel box and the particles were grouped by reference-free 2D classification. Classes with contamination and damaged particles were discarded and the remaining particles were aligned on a reference generated from the PDB entry 5G5L low-pass filtered to 40 Å. Three-dimensional (3D) classes containing only Pol I and Rrn3, or damaged particles were discarded. The remaining 227,718 particles from the four subsets were merged, re-extracted without binning and refined against an initial model generated in RELION. CTF Refinement and Bayesian polishing was performed and the polished particles were refined and 2D and 3D classification without alignment were performed to remove misaligned particles and the remaining 168,532 particles were subjected to a second round of CTF refinement. A 3D classification without sampling and a CF-only mask revealed one class with partial CF occupancy and another with damaged particles that were both discarded. Refinement of the remaining 122,099 particles resulted in an early intermediate PIC reconstruction. For details, compare Supplementary Fig. 1. During post-processing in RELION, a B-factor of −75 Å² was determined and applied for map sharpening, resulting in an overall resolution of 3.5 Å. Focused refinements the with a Pol-I-Rrn3 mask (3.5 Å after post-processing) or a CF-DNA mask (3.9 Å after post-processing) were additionally carried out to assist subdomain conformation determination and aid CF chain tracing, respectively. Directional FSC were calculated as described^80^.

### Model building

At a resolution of 3.5 Å, we derive an atomic model of an early intermediate PIC. We first placed Pol I domains as described for PDB 5G5L^12^ originating from the crystal structure (PDB 4C2M^30^), an Rrn3 monomer (PDB 3TJ1^32^), a CF monomer (PDB 5O7X^15^) and an ITC DNA (PDB 5W66^17^) in the unsharpened eiPIC map generated with RELION 3 (beta version)^33^. Using COOT^81^, we adjusted protein backbone traces consulting focused maps of CF or the Pol-I-Rrn3 complex and finally build side chain residues where appropriate. DNA-sequences were mutated to poly-A (-T, -G, -C). For the structure-based modeling of the TFIIB-related domains in the N-terminal region of Rrn7, the strong density for aromatic residue Phe70 was used as a marker. The final model was refined using the real-space refinement tool of the Phenix suite^82^ and evaluated using MolProbity^83^. Figures were prepared with UCSF Chimera^84^ or PyMOL (pymol.org).

It should be noted that promoter-binding regions within CF are highly flexible and thus poorly ordered in DNA-free CF crystals^15^. While we refrained from building most of these regions in the crystal structure, the putatively assigned residue numbers within helix α2 of CF subunit Rrn11 were now adjusted in the eiPIC, similar to a de novo built model based on a cryo-EM reconstruction of an ITC^17^.

An additional cryo-EM density stretch between the Rrn7 ribbon and the Pol I wall domain may potentially be attributed to a flexible loop in Rrn3 (249–323) or to a part of the Rrn6 C-terminal domain. Whereas the latter assignment would agree with a previously published crosslinking/mass spectrometry analysis^16^ and direct Rrn6-Rrn3 interaction studies^85^, it remains as speculative at this point.

### Reporting summary

Further information on research design is available in the Nature Research Reporting Summary linked to this article.

## Supplementary information

Supplementary Information

Reporting Summary

## Data Availability

The cryo-EM density of the Pol I eiPIC has been deposited in the Electron Microscopy Data Bank under accession code EMD-10544 and coordinates of the eiPIC model have been deposited with the Protein Data Bank under accession code 6TPS. Focussed refinement density of CF has been deposited under EMD-10663. Other data are available from the corresponding author upon reasonable request.
