# Supplementary material for: Structural basis of RNA polymerase I pre-initiation complex formation and promoter melting
Source: Nat Commun. 2020 Mar 5;11:1206. doi: 10.1038/s41467-020-15052-y (PMC7057995; doi:10.1038/s41467-020-15052-y)
Supplement: Supplementary file 1 — Supplementary Information [file 41467_2020_15052_MOESM1_ESM.pdf]

Supplementary Information

Structural basis of RNA polymerase I pre-initiation complex formation and promoter melting

Michael Pilsl and Christoph Engel

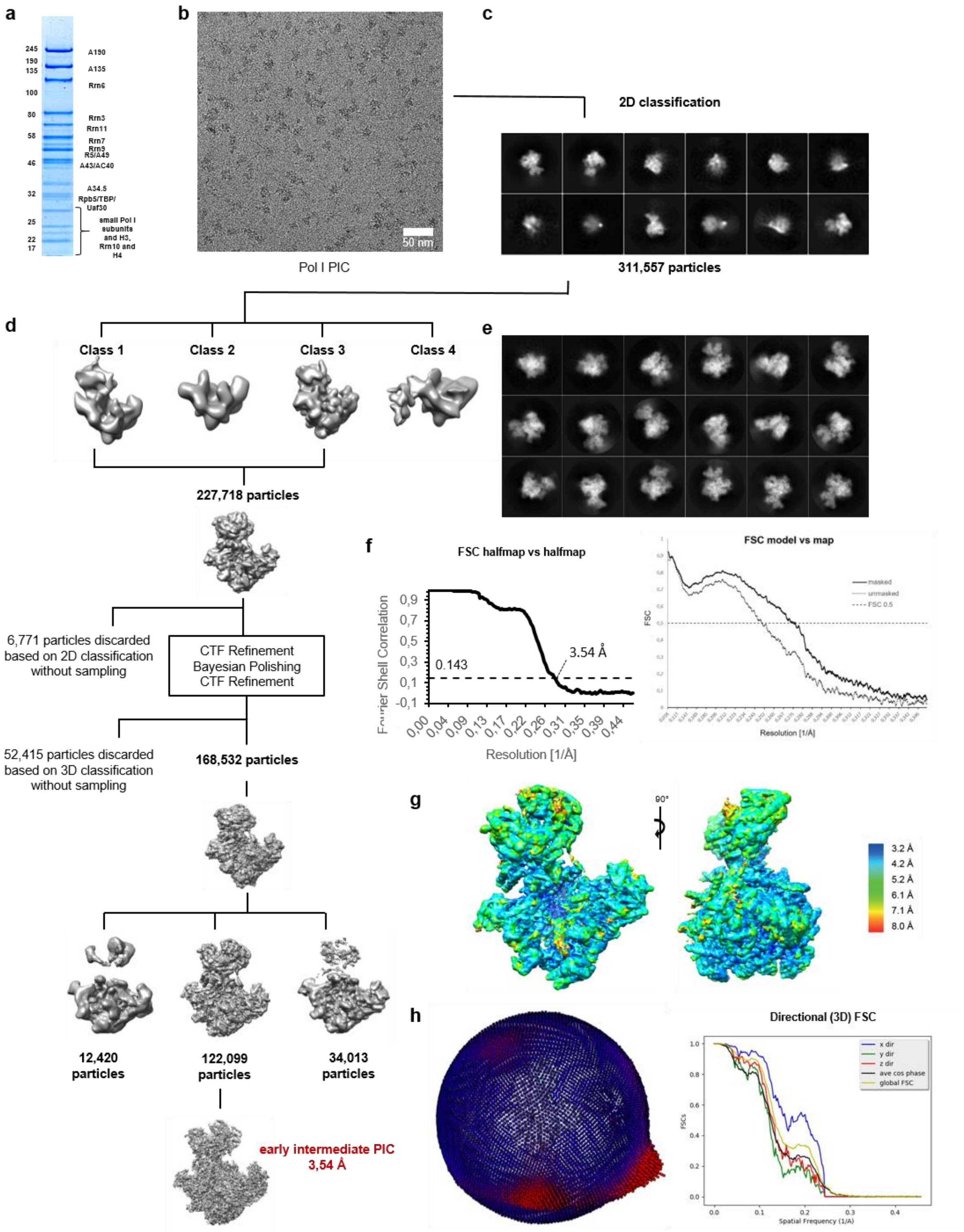

Supplementary Figure 1. Formation and cryo-EM processing of eiPIC.

a Coomassie-stained SDS-PAGE of non-crosslinked PIC samples shows the presence of all polypeptide chains in approximately stoichiometric amounts. **b** Representative raw cryo-electron micrograph. **c** Representative reference-free 2D class averages of initially extracted particles. **d** 3D classification of the Pol I PIC data set (Methods). The particles were subjected to a hierarchical process, which encompassed several rounds of classification. The number of particles contributing to each class is indicated. First round of 2D and 3D classification was performed with four subsets and threefold binning. **e** Representative 2D class averages of particles in final reconstruction. **f** Fourier shell correlation of eiPIC half-datasets with the estimated resolution at the gold-standard FSC (FSC = 0.143) and model vs. map FSC. **g** Local Resolution estimation. **h** Orientational distribution of the particles contributing to the eiPIC reconstruction, left panel: height of the surface bars indicates the relative number of particles in a given orientation, right panel: directional FSC (compare Methods).

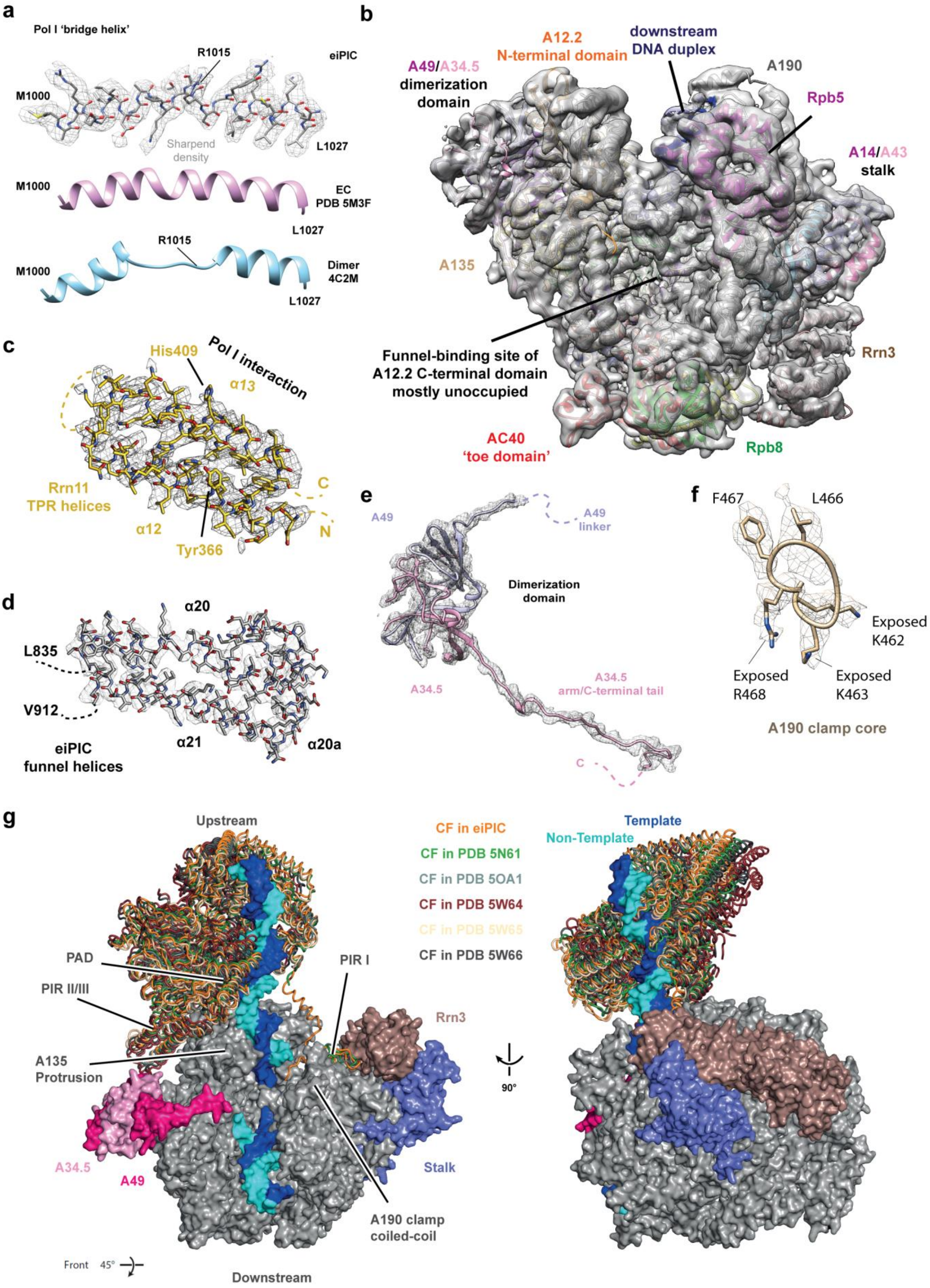
a
Pol I 'bridge helix'
R1015
eiPIC
M1000
Sharpend density
L1027
M1000
EC PDB 5M3F
L1027
R1015
M1000
Dimer 4C2M
L1027
b
A12.2 N-terminal domain
downstream DNA duplex
A49/A34.5 dimerization domain
A190
Rpb5
A14/A43 stalk
A135
Funnel-binding site of A12.2 C-terminal domain mostly unoccupied
Rrn3
AC40 'toe domain'
Rpb8
c
His409
Pol I interaction
α13
Rrn11 TPR helices
α12
Tyr366
C
N
d
α20
L835
V912
eiPIC funnel helices
α21
α20a
e
A49
A49 linker
Dimerization domain
A34.5
A34.5 arm/C-terminal tail
C
f
F467
L466
Exposed K462
Exposed R468
Exposed K463
A190 clamp core
g
Upstream
CF in eiPIC
CF in PDB 5N61
CF in PDB 5OA1
CF in PDB 5W64
CF in PDB 5W65
CF in PDB 5W66
Template
Non-Template
PAD
PIR II/III
PIR I
Rrn3
A135 Protrusion
90°
A34.5
A49
Stalk
A190 clamp coiled-coil
Front 45°
Downstream

Supplementary Figure 2. Cryo-EM density and factor occupation of eiPIC reconstruction.

a Central bridge helix is refolded in eiPIC reconstruction. Sharpened eiPIC density (Focused on Pol I – Rrn3) is depicted as grey mesh and superimposed with atomic model. Models of completely folded bridge helix in a Pol I EC (pink, PDB 5M3F) and the unfolded helix in Pol I dimers (light blue, PDB 4C2M) shown for comparison. **b** eiPIC cryo-EM density (grey, transparent envelope) superimposed with the eiPIC structural model indicates low to no evidence of A12.2 C-terminal domains presence. **c** Rrn11-TPR density is depicted as grey mesh and superimposed with atomic model. **d** Atomic model of the funnel helices within Pol I subunit A190 overlaid with sharpened eiPIC density (grey mesh). **e** eiPIC density (grey mesh) shows a well-defined A49/A34.5 sub-complex (ribbon). **f** Exposed positive charges in A190 clamp core, atomic model overlaid with sharpened eiPIC density (grey mesh). **g** Location of CF in Pol I initiation complexes. 10-subunit core of Pol I is shown in gray with the A14/A43 stalk sub-complex in slate, Rrn3 in dark red and the A49/A34.5 dimerization domain in pink (space filling). PDB models were superposed via the subunit A135. The CF location is similar in all structures as shown by ribbon models of the subunits Rrn6, Rrn7, and Rrn11 in orange eiPIC, green (5N61), tin (5OA1), brown (5W64), wheat (5W65), and dark grey (5W66).

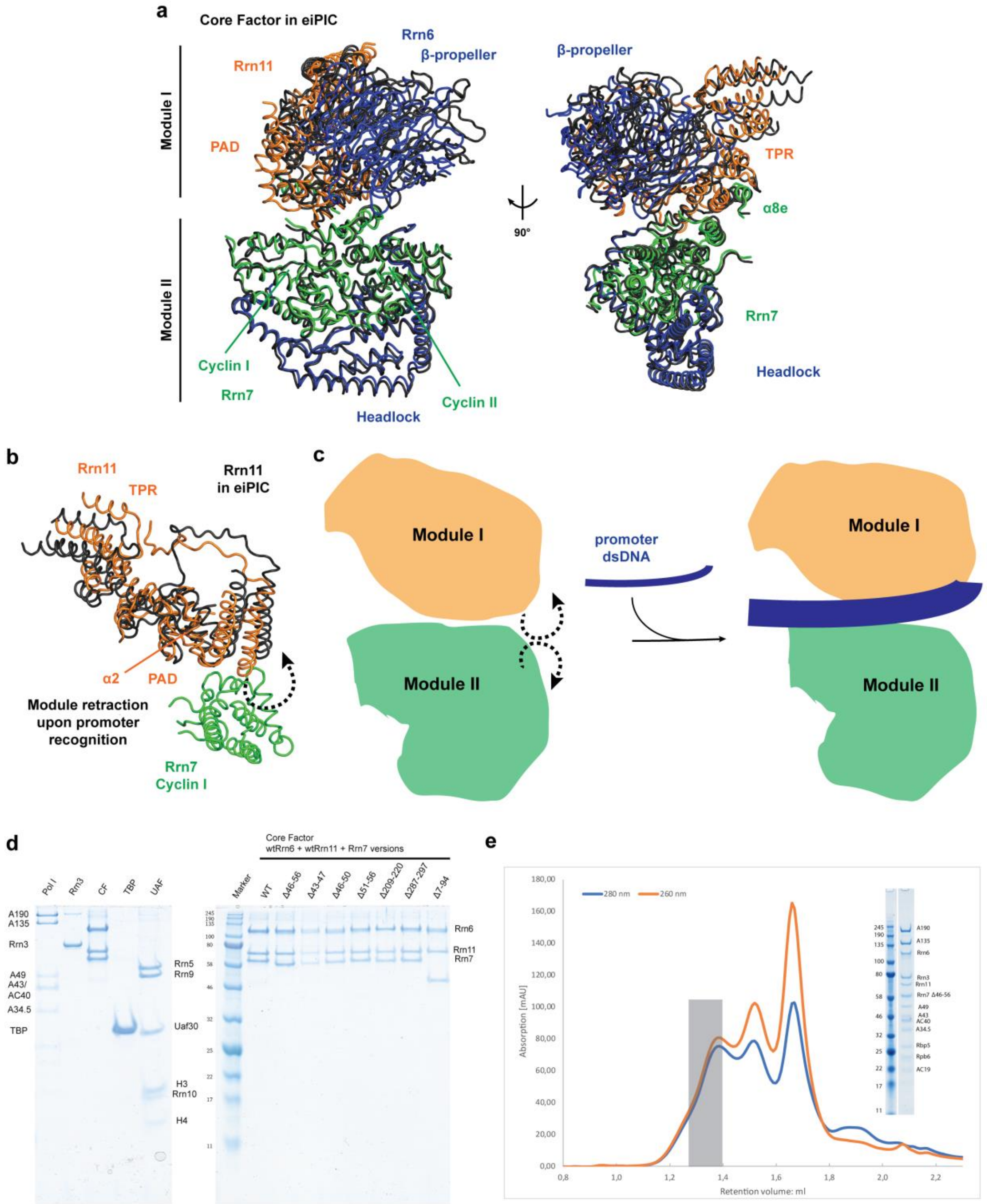

Supplementary Figure 3. Core Factor modules retract upon promoter recruitment.

a Structure of free CF (PDB 5O7X; subunits Rrn6, Rrn7 and Rrn11 in blue, green and orange, respectively) overlaid with promoter-bound CF (black) via their Rrn7 cyclin domains. **b** CF modules retract from each other to expose phosphate-backbone-interacting regions (Colors as in a). **c** Schematic representation of CF module retraction upon DNA binding, module I depicted in orange, module II in green and DNA in blue. Arrows with dotted line indicate movement of modules. **d** Coomassie-stained SDS-PAGE shows the used complexes, single proteins and CF-mutants for comparison. **e** SEC of CF mutant version with deleted Rrn7 B-reader loop. The mutant still assembles with Pol I and Rrn3 on promoter DNA. Right panel: Coomassie-stained SDS-PAGE of marked elution fraction.

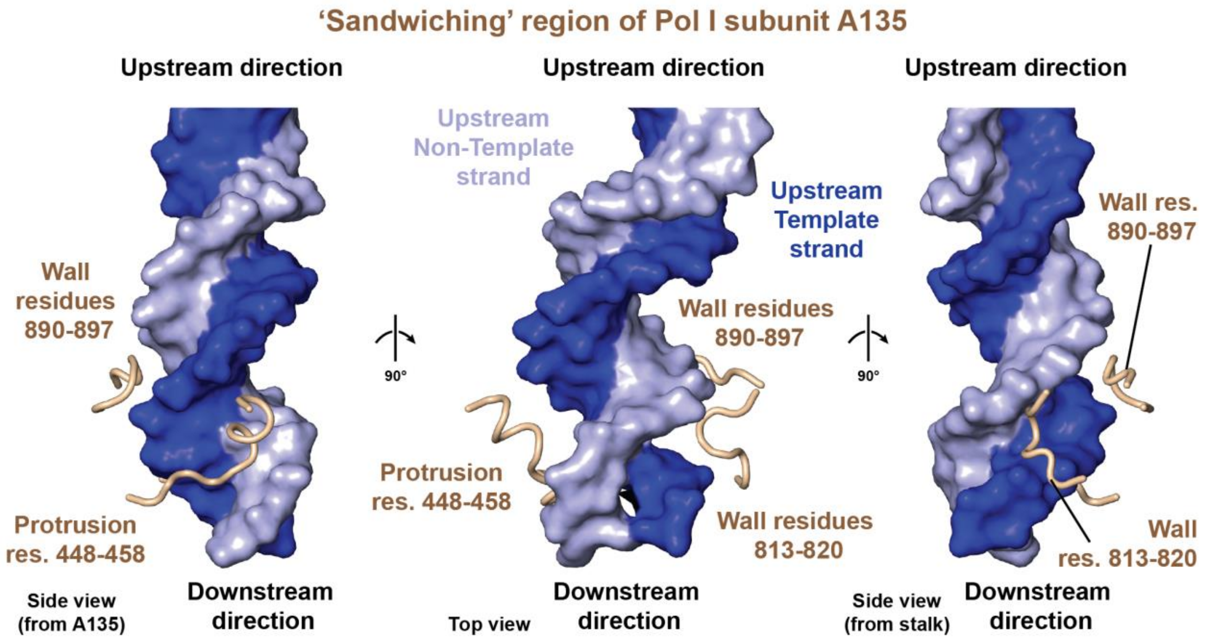

Supplementary Figure 4. Pol I - specific loops within subunit A135 form a 'sandwich' region.

PDB model of proximal upstream promoter DNA in the eiPIC (space-filling, blue) shows how A135 residues (ribbon, wheat) approach the backbone of both DNA strands.

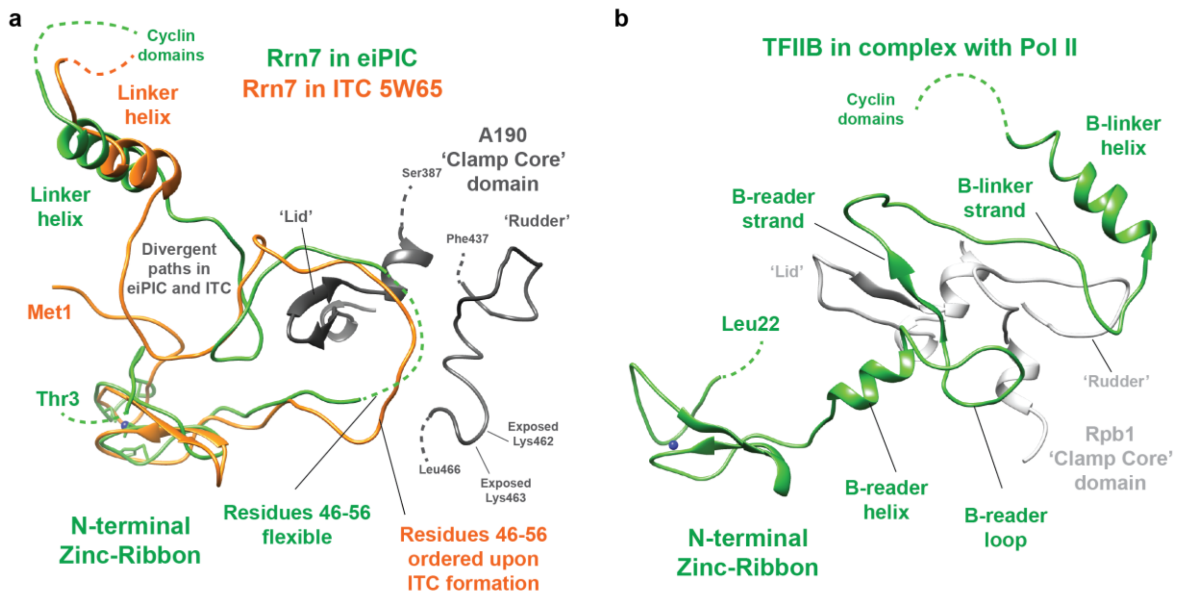

Supplementary Figure 5. Rrn7 path in the Pol I cleft.

a Structure of Rrn7 in the eiPIC (green) compared to the ITC (PDB 5W65, orange). While the residues 46 to 56 are disordered in the eiPIC, they are mostly structured in the ITC. Divergent chain traces upon Pol I exit may indicate different stages of Rrn7 action, they may also result from different interpretation of densities (compare Fig. 3). **b** For comparison, the path of TFIIB in an ITC crystal structure is indicated (PDB 4BBS, green).

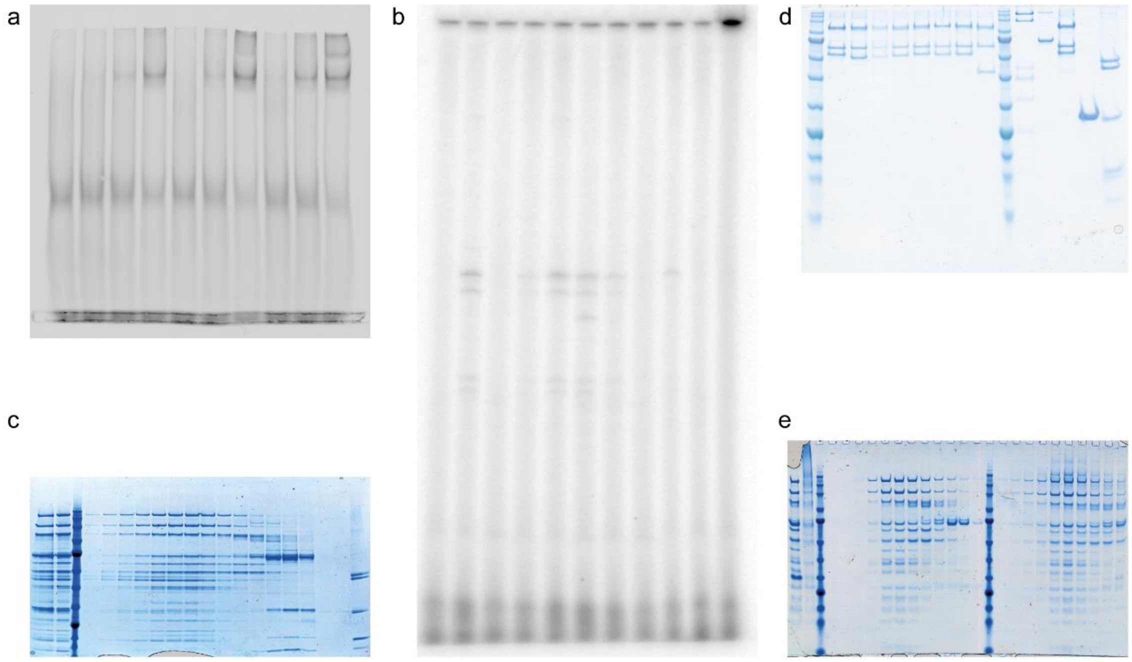

Supplementary Figure 6. Raw Gel images.

a Native PAGE/EMSA as shown in Fig. 2b and described in methods. **b** Autoradiogram UREA-PAGE of promoter-dependent *in vitro* transcription by CF mutants as shown in Fig. 2c and Fig. 3b. **c** Coomassie-stained SDS-PAGE as shown in Supplementary Figure 1a. **d** Coomassie-stained SDS-PAGE as shown in Supplementary Figure 3d. **e** Coomassie-stained SDS PAGE as shown in Supplementary Figure 3e.

Supplementary Table 1. DNA oligonucleotides used in this study.

TBP cloning	
ID 4224	TTTTGCTAGCATGGCCGATGAGGAACGTTT
ID 4225	TTTTGCGGCCGCTCACATTTTTCTAAATTCACTTAG
UAF cloning	
ID 2334	TTTTTTCTCGAGATGGCCAGAACAAAGCAAAC
ID 2335	TTTTTTGCTAGCCTATGATCTTTCACCTCTTA
ID 2336	TTTTTTGGATCCATGTCCGGTAGAGGTAAAGG
ID 2337	TTTTTTTCTAGATTAACCACCGAAACCGTATA
ID 2338	TTTTTTGGATCCATGGATAGAAATGTATATGA
ID 2339	TTTTTTTCTAGATCAGATATTACCTGGCGCAT
ID 2340	TTTTTTCCCGGGATGGAGCACCAACAATTGCGGAAGT
ID 2341	TTTTTTGCTAGCGCGCGCCCTAGCACTGAGCAG
ID 2342	TTTTTTCTCGAGATGAGTGATCTTGACGAAGA
ID 2344	TTTTTTGCTAGCTCACATCGTCGTCATCCTTGTTTCCCATTAGGCAGTT
ID 2345	TTTTTTGGATCCATGGCTGAATTAAACGATTA
ID 2346	TTTTTTAAGCTTTTTAGAAGTGGCGCGCCCTA
Rrn7 mutagenesis	
ID 4668	TTAACGACGATGAAACAAGACGTCTGAATCTTACCACCAATGC
ID 4669	TTCAGACGTCTTGTTTCATCGTCGTTAAATTCCACGTCACCTT
ID 4670	AGGTGACGTGGAATTTAACCTCAACGGCCTCGG
ID 4671	CCGAGGCCGTTGAGGTTAAATTCCACGTCACCT
ID 4672	TTAACGACGATGAACTCGGTGCAGGTGTTATCACAAGACGTC
ID 4673	AACACCTGCACCGAGTTCATCGTCGTTAAATTCCACGTCACCTTCCATAACG
ID 4674	ATGATCTCAACGGCACAAGACGTCTGAATCTTACCACCAATG
ID 4675	TTCAGACGTCTTGTGCCGTTGAGATCATCTTCATCGTCG
ID 4676	GATCCAGCTGCCACCTTTTAATGGGCAACTGTACAACAAAATCG
ID 4677	CCCATTAAAAGGTGGCAGCTGGATCCTCCACGATTTCGGTAGT
ID 4678	GAACCTGACACTGGTAAGTAATCATGCCGAACTCAGGGTCCTCTC
ID 4679	CATGATTACTTACCAGTGTCAGGTTCCGAATGTCTGTTTCTTCAAATTCGATAAC
Promoter amplification	
ID 4685	AGCTTAAATTGAAGTTTTTCTCGGC
ID 4686	TCGAACTTGTCTTCAACTGCTTTC
ID 4688	TTTCGCATGAAGTACCTCCCAAC
Synthetic promoter constructs	
ID 4689	GAGTACAAGTGTGAGGAAAAGTAGTTGGGAGGTACTTCATGCGAAAGCAGTTGAAGACAAG
ID 4690	CTTGTCTTCAACTGCTTTCGCATGAAGTACCTCCCAACTACTTTTCCTCACACTTGTACTC
ID 4691	AAAAGTAAGATTTTAGTTTGTAATGGGAGGGGGAATTTAGTCATGGAGTACAAGTGTGAGG<br>AAAAGTAGTTGGGAGGTACTTCATACCAAAGGGGTTCAACACAAGGGT[ATTO647N]
ID 4692	ACCCTTGTGTTGAACCCCTTTGGTATGAAGTACCTCCCAACTACTTTTCCTCACACTTGTA<br>CTCCATGACTAAATTCCCCCTCCCATTACAAACTAAAATCTTACTTTT[CY3]
ID 4693	AGCTTAAATTGAAGTTTTTCTCGGCGAGAAATACGTAGTTAAGGCAGAGCGACAGAGAGGGCAAAAGAAAATAAAAGTAAGA<br>TTTTAGTTTGTAATGGGAGGGGGGGTTTAGTCATGGAGTACAAGTGTGAGGAAAAGTAGTTGGGAGGTACTTCATGCGAAA
ID 4694	TTTCGCATGAAGTACCTCCCAACTACTTTTCCTCACACTTGTACTCCATGACTAAACCCCCCCTCCCATTACAAACTAAAAT<br>CTTACTTTTATTTTCTTTTGCCCTCTCTGTCGCTCTGCCTTAACTACGTATTTCTCGCCGAGAAAAACTTCAATTTAAGCT
